# 3D Measurement of Large Deformations on a Tensile Structure during Wind Tunnel Tests Using Microsoft Kinect V2

**DOI:** 10.3390/s22166149

**Published:** 2022-08-17

**Authors:** Daniele Marchisotti, Paolo Schito, Emanuele Zappa

**Affiliations:** Department of Mechanical Engineering, Politecnico di Milano, 20156 Milano, Italy

**Keywords:** wind tunnel, Kinect V2, 3D measurements, 3D reconstruction, point cloud registration

## Abstract

Wind tunnel tests often require deformation and displacement measures to determine the behavior of structures to evaluate their response to wind excitation. However, common measurement techniques make it possible to measure these quantities only at a few specific points. Moreover, these kinds of measurements, such as Linear Variable Differential Transformer LVDTs or fiber optics, usually influence the downstream and upstream air fluxes and the structure under test. In order to characterize the displacement of the structure not just at a few points, but for the entire structure, in this article, the application of 3D cameras during a wind tunnel test is presented. In order to validate this measurement technique in this application field, a wind tunnel test was executed. Three Kinect V2 depth sensors were used for a 3D displacement measurement of a test structure that did not present any optical marker or feature. The results highlighted that by using a low-cost and user-friendly measurement system, it is possible to obtain 3D measurements in a volume of several cubic meters (4 m × 4 m × 4 m wind tunnel chamber), without significant disturbance of wind flux and by means of a simple calibration of sensors, executed directly inside the wind tunnel. The obtained results highlighted a displacement directed to the internal part of the structure for the side most exposed to wind, while the sides, parallel to the wind flux, were more subjected to vibrations and with an outwards average displacement. These results are compliant with the expected behavior of the structure.

## 1. Introduction

Wind tunnel tests usually investigate the response of structures to wind in terms of deformation and displacement [1]. For these tests, it is essential to measure deformations and displacements on a large number of points on a structure, for a comparison with mathematical models [2]. In addition, measuring the 3D shape and deformations of an entire structure could be more suitable for simulation model comparison, since usually mathematical models refer to the entire structure [3], while traditional measurement techniques can often return data referred to a few points (e.g., Linear Variable Differential Transformers LVDTs, fiber optics, and strain gauges).

Displacement measures could be performed using common and traditional approaches and instruments. For example, the application of LVDT sensors or laser doppler vibrometers [4] can be helpful also for determining natural frequencies, as well as for time domain analysis. In addition, the application of fiber optics [5] can return small deformation measurements at specific points and they are also commonly used for structural monitoring [6]. However, these techniques can be invasive for wind tunnel tests [3]. For example, LVDT sensors have to be placed in direct contact with the surface for measuring displacement, changing the wind flow, and affecting the test. Fiber optics, accelerometers, and pressure sensors are less intrusive, but, if it is necessary to determine the shape of an object, it will be difficult to acquire many different sensor signals, leading to a quite complex setup and acquisition system. Moreover, they should be applied to the surface to be measured leading to possible changes in its properties.

With these considerations, non-contact measurement techniques can be addressed as less invasive measurement methods. In this sense, laser doppler vibrometers could be used for wind tunnel tests [7], but they could interfere with the air flux and they could have a limited Field of View (FOV) and measurement range. In addition, the application of a vibrometer could disturb the wind flux. Therefore, to obtain the shape of an object during wind tunnel tests, a sensor with a large FOV, to be placed far away from the structure, and can return a point cloud at a specific time, can be of great advantage in the case of measurements of entire object displacements. 

Within this framework, in this article, the application of 3D Time-of-Flight (ToF) cameras, such as Kinect V2, is investigated in wind tunnel tests, for 3D shape measurement, with the objective of measuring the structure displacement for different wind velocities. For this purpose, the application of 3D cameras returning a point cloud for a specific time instant can help to obtain the entire 3D shape under different testing conditions. Traditional optical measurement techniques can be used as well [8,9], but they would involve the application of markers or spraying patterns [10], leading to a modification of the model surface. In the case of reflective markers consisting of solid spheres, object surfaces could be modified, and the response of the structure to wind would be different. Three-dimensional laser scanners, such as the ones used in [11,12], could be used as well, but they usually require a scanning time of a few seconds to complete a scan and if the object to measure is subjected to vibration, the final result will be affected by noise. In addition, these techniques have a limited measurement range and a limited FOV, while the proposed method could perform measurements in a volume of a few meters, it is possible to place the sensors close to the walls of the wind tunnel chamber, reducing the effect on the downstream and upstream wind fluxes. The proposed method is one of the less expensive wind tunnel tests for 3D measurements. For these reasons, it would be difficult and not very meaningful to perform a comparison with the cited techniques, which are more expensive and have very different characteristics. In this paper, the feasibility of the application of 3D ToF cameras, such as Kinect V2, is studied, since they represent a valid option for their compactness, lightness, and low cost and, to the best of our knowledge, are not present in the literature. At the same time, they do not require markers and they can be used for 3D shape measurement to obtain the displacement of the entire structure. In addition, the greater measurement range, compared to the devices mentioned in this paragraph, permits the sensors to be placed close to the walls of the wind tunnel chamber, reducing the effect on the downstream and upstream wind fluxes. The measurement method is based on:

1. Calibration of the 3 Kinect V2 placed in the wind tunnel to align all the three sensors to a unique reference system (Section 3.3);
2. Extraction of sections of the structure measured to identify the displacement on those sections for different wind velocities (Section 3.5);
3. Subdivision of each section into different sides of the object (Section 3.5);
4. Definition of the undeformed object sections given by fitting 3D reconstructions obtained from undeformed object acquisitions (Section 3.5);
5. Calculation of the displacement for each side provided as the average and standard deviation displacement, in relation to the undeformed condition, along each side coordinate (Section 3.5);
6. Evaluation of the average displacement of each side to obtain more compact data (Section 4).

The paper is structured as follows: in Section 2, a literature review is presented to introduce the scientific context of the article; in Section 3, an experimental setup is described; in Section 3, the sensors point cloud registration and the data analysis method are explained; test results are presented and discussed in Section 4; and in Section 5, conclusions about the test method and results are drawn.

## 2. Related Works

In the literature, vision-based measurements may require the presence of markers or at least features to track in order to obtain the displacement of an object on the image plane [13]. On the other hand, 3D scanners can project infrared or visible light patterns, whose reflection can be used to obtain depth information. Using this method, 3D scanners do not require the presence of markers to obtain a set of points in a 3D environment and to perform displacement measurements for monitoring, e.g., monitoring of human breathing [14]. In particular, Time-Of-Flight (ToF) devices, such as Kinect V2, project IR light on the entire scene and measure the elapsed time between the light source emission and reception after its reflection on a target. This time delay is determined for Kinect V2 as the reflected energy and sampled at every pixel, using two windows with a phase shift of 180° (pulsed modulation) [15]. In this way, it is possible to perform wind tunnel tests without interfering with the aerodynamical properties of the structure under test. Moreover, it was also demonstrated that the application of depth cameras, in wind tunnel tests, is suitable to estimate the pose of an object subjected to wind, as addressed in [16], or to determine the position and orientation of an ultra-small airplane [17]. Indeed, pose estimation is one of the main applications of low-cost depth cameras, such as Kinect V2, using also multiple cameras [18], and also for cameras covered by a protective glass [19].

Regarding the 3D measurement applications, the main limitation of depth cameras is their uncertainty of a few millimeters [20], when it is required to measure objects with high stiffness and subjected to submillimeter displacement. For these conditions, it could be more appropriate to use one single camera or stereo cameras with a high-quality sensor and high framerate to perform Digital Image Correlation (DIC) [21]. This technique can be used for measuring strains of mechanical components, also using 3D-DIC [22], also in a wind tunnel environment [23]. However, using these techniques, it is possible to measure only a reduced part of an object to perform DIC effectively, with speckle reproduced on the object. On the other hand, further studies presented in the literature involve the application of photogrammetry for the 3D shape measurement of large offwind yacht sails, which were also carried out in wind tunnels [24,25]. As explained in [24], the precision of this measurement system is of few centimeters, which is not significant for measuring the apparent wind angles (AWA) and spinnaker shapes, but it can be important for other objects’ wind tunnel tests. Moreover, for 3D reconstructions in a wind tunnel, more than one camera or stereo camera (four cameras in [24]) can be required, leading to a quite expensive and cumbersome setup. On the contrary, the accuracy can significantly decrease in the case of the application of Light Detection and Ranging (LIDAR) sensors, as in [26]. However, in this case, the measurement system is based on a custom sensor, which can be quite expensive in terms of instrumentation and involves considerable work to set up the system. As for the system presented in this article, if the displacement of a structure, such as the one used for the test presented in this article, is much higher than the uncertainty of the Kinect V2 sensor, the application of our measurement method is a valid option for measuring 3D displacements without advance equipment and with a more user-friendly acquisition. In particular, the uncertainty of Kinect V2 is 1.2 mm at about 1500 mm to 3.3 mm at the maximum reliable distance (4200 mm) [20], (much lower than photogrammetry measures), while the displacement of the structure under test was estimated to be of few centimeters. At the same time, the wind tunnel chamber used for the test is 4 m × 4 m × 4 m; thus, Kinect V2 can measure inside its measurement range, if the object to measure is placed at the center of the chamber. Thus, in this case, the application of depth cameras is suitable for this test.

In the literature, 3D reconstruction of a civil structure could be performed with different state-of-the-art techniques. These algorithms for obtaining a 3D shape reconstruction usually involve the movement of a camera around the structure to capture a set of 2D images to be processed using Structure from Motion (SfM) algorithms [27] or a set of RGBD images to be processed using Simultaneous Localization And Mapping (SLAM) algorithms [28]. However, during wind tunnel tests, it is not possible to change the position of a 3D scanner and the structure is not still during tests; therefore, these algorithms would not be able to obtain reliable results. For these reasons, in this research, the application of multiple Kinect V2 sensors for 3D reconstruction is presented with the specific application of 3D displacement measurement, for wind tunnel tests, without interference from the upstream and downstream wind and with calibration of the system executed directly in the wind tunnel.

## 3. Materials and Methods

The experimental setup is composed of two parts. The first one is defined by the structures to be tested and the second is the optical measurement system.

### 3.1. Tensile Structures

**Figure 1:**
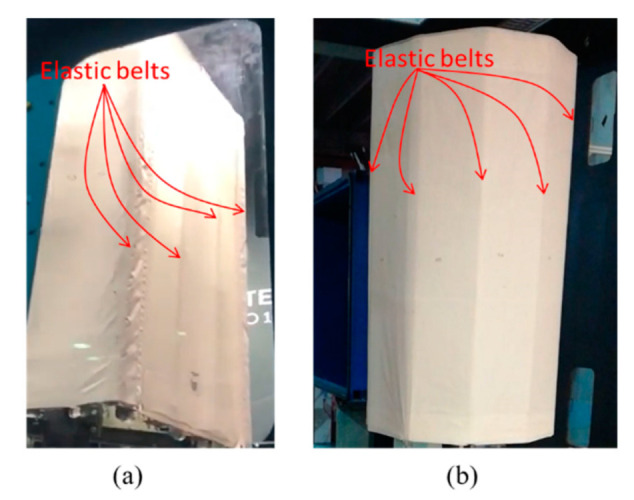
Squared (**a**) and decagonal (**b**) structures under test.

**Figure 2:**
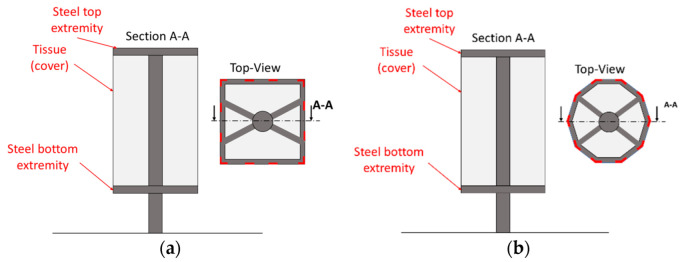
A schematic of the squared (**a**) and decagonal (**b**) structures under test. The positions of elastic belts are highlighted by using red lines, in the top view.

For the wind tunnel test, the structures under test were based on a squared and a decagonal base prism, respectively (Figure 1). The two structures are based on a tissue (cover) made of polyester micro-pierced tissue, which is the part being evaluated and the one more subjected to greater displacement. The cover is linked to the steel structure only at the top and bottom extremities (referred to as steel to extremity and steel bottom extremity in Figure 2) and it can be easily deformed by a few centimeters by hand. This link between the cover and the steel extremities is obtained with glue and small screws to prevent them from fluttering, creating a disturbance to the flow and leading to potential local damage of the cover tissue. The tissue displacement under the effect of the wind is much higher than the state-of-the-art uncertainty of ToF depth cameras. The height of both structures is 3500 mm and the cover starts from the bottom steel extremity at 580 mm from the wind tunnel floor. For the squared structure, the side length is 1200 mm, and for the decagonal structure, the diameter of the circle containing the prism base is 1230 mm. The tensile structure is made out of micro-pierced polyester tissue. This cover is connected at the extremities to a steel border base and the same border is replicated at the top of the structure. The steel structure is connected to a central steel column. A scheme of both structures is visible in Figure 2. To reinforce the central part of the structure, elastic belts connect the two borders, constraining the cover to a smaller displacement. Elastic belts are highlighted in Figure 1 using red lines in the top view of the schematic in Figure 2. In Figure 2, the belts are represented as short lines to schematically represent that they are attached to the steel extremities and do not cross the top and bottom base of the structures. The cross section of the elastic belts is 60 × 5 mm and the length of the elastic belts is equal to the height of the cover. The cover thickness is 1 mm. This tissue could be broken by wind or it could be plastically deformed if it is not reinforced by elastic belts; this is the reason for the presence of elastic belts. A side is equipped with a small entrance to access the internal part of the structure. This side is considered as the door side. The entire structure was fixed on a rotating plate to make it possible to perform tests for different wind directions. The two described structures are shown in Figure 1 and Figure 2. The two structures, squared and decagonal, are simple structures whose displacements can be measured using the proposed technique, since the displacement of the cover is much higher than the Kinect V2 measurement uncertainty. This measurement method can also be extended to more complex structures with known geometry. There is no particular reason why these two structures were selected. They simply represent a case study of the measurement method.

### 3.2. Measurement System

The measurement system is based on 3 Kinect V2 3D cameras used to reconstruct the 3D geometry of the two structures with the main goal to determine the average displacement of the object subjected to different wind velocities.

For our test, among the different technologies and sensors available on the market, Kinect V2 was chosen, since its characteristics (Table 1) are compatible with the requirements of the test. In particular:

1. Total measurement range: 0.7–4.2 m [20];
2. Displacement of the tensile structure, which is a few centimeters and it is much higher than the random error of the Kinect V2 sensor, which is 1.2 mm at about 1500 mm to 3.3 mm at the maximum reliable distance (4200 mm) [20];
3. Low latency time of 20 ms to acquire a depth image [20];
4. Absence of markers, which could affect or damage the structure during the test;
5. The structure is convex; thus, the multiple reflection errors that are typical of Time-Of-Flight devices are not present [29];
6. Low cost: during wind tunnel tests many cameras could be required and their usage could be limited to a few tests. Thus, large investments for very low uncertainty devices could be not justified.

“Max Depth Resolution” and “Max Color Resolution” in Table 1 are the resolutions of the depth sensor and RGB camera of Kinect V2. They are referred to as max since it is possible to acquire depth and color images with a lower resolution by changing the acquisition mode of the sensor. In the current work, the maximum resolution is used.

**Figure 3:**
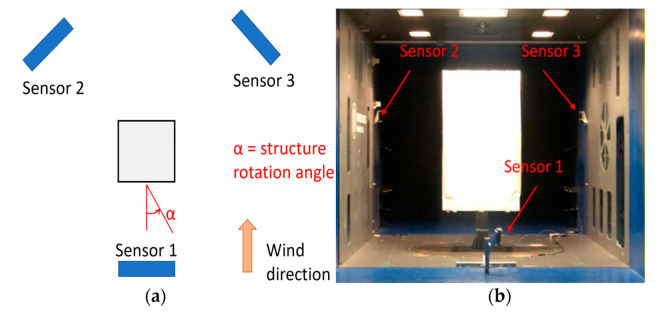
Scheme of the sensor positions (**a**) and actual positions (**b**).

Since it is not possible to move Kinect V2 around the structure during the test to perform the 3D reconstruction in a conventional way, 3 Kinect V2 sensors were placed according to the scheme in Figure 3. The number of sensors was limited to the minimum, since a higher number of sensors would not significantly reduce the uncertainty, unless a very high number of sensors was applied with a high overlap of point clouds, to have a large number of measurements at the same point.

**Figure 4:**
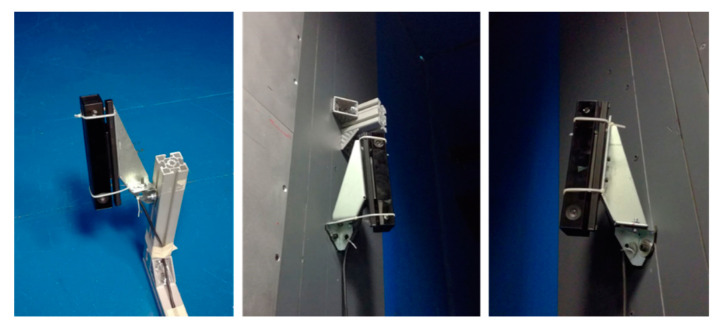
Pictures of the Kinect V2 1, 2, and 3, respectively, for wind tunnel test (cable ties to fix the sensors do not affect the point cloud acquisition).

Sensor 1 was placed at a height of about 0.2 m from the ground and it was inclined with respect to the vertical direction at about 20° (Figure 4). On the other side, sensors 2 and 3 are connected to the walls of the wind tunnel at a height of about 2.5 m and rotated toward the structure and downwards. All sensors were installed with their longer dimension close to the vertical direction to exploit the higher FOV of Kinect V2 on the horizontal direction (Table 1). In this way, the structure is centered on the depth image of each sensor, limiting the errors since it was demonstrated that close to the borders of the depth image, the Kinect V2 sensor is less precise, due to the projection IR cone that is less intense going to the corners [20].

The acquisition of data from sensors was performed using *libfreenect2* API [30]. This API permitted us to acquire up to 5 sensors simultaneously by using the same PC, instead of the *Kinect for Windows SDK2.0,* for which only one single device acquisition is permitted. The synchronization of devices was not required for our case, since the average and standard deviation of displacement do not require particular synchronization of devices. The average and standard deviation of displacement were computed for specific sections of the structure, with reference to the sides of the structure, as described in Section 3.5.

### 3.3. Measurement System Calibration: Point Cloud Registration

Before starting the test, it is necessary to calibrate the vision system. This procedure is performed to find those transformation matrices to align the 3 Kinect V2 sensors with respect to the same reference system. Then, point clouds from the 3 sensors are registered in post-processing to obtain the full 3D geometry of the structure for each frame acquired, assuming the 3D cameras are not moving during the test. This assumption is based on the consideration that the wind force acting on a single sensor, in the worst case, was estimated to be 20–30 N, while sensors were fixed using cable ties able to resist a force of about 10 times higher. 

Calibration was performed to ensure that the transformations aligned with the point clouds from sensors 1 and 2 to sensor 3. To obtain this result, a thin wooden plane was used in order to acquire the point cloud from all 3 sensors at the same time in static conditions, and the point clouds obtained from the sensors were segmented to extract the plane. To better identify the plane, high-reflective tape was put on the corners of the plane since it can lead to out-of-range measures for ToF cameras, such as Kinect V2 [29]. Using this method, it was easier to find the corners of the plane and to extract the part of the point cloud related to the plane. All the 3 point clouds representing a single plane were then aligned with the Iterative Closest Point (ICP) algorithm [31], and the transformation obtained matrices were referred to the one of sensor 3. To consider the 10 mm thickness of the wooden plane of the point clouds from sensor 1, the extracted point cloud was modeled by using a plane, and then the point cloud was translated 10 mm along the normal direction of the plane. 

**Figure 5:**
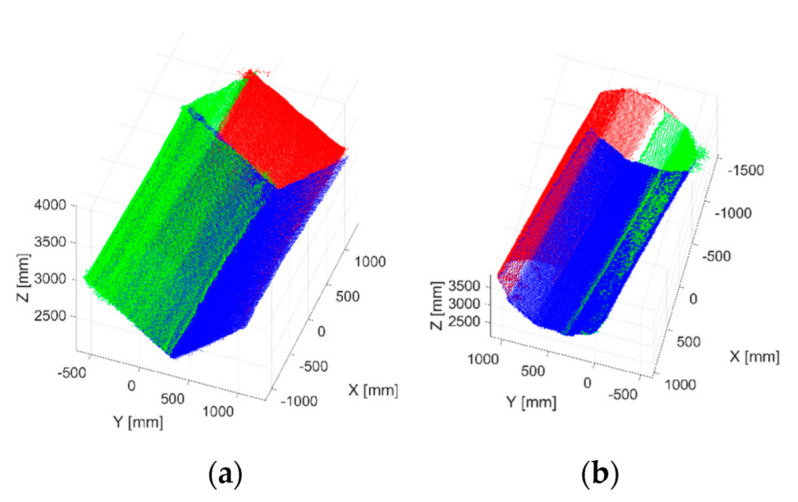
Three-dimensional reconstruction of the rectangular (**a**) and decagonal (**b**) tensile structures example (different colors refer to point clouds acquired from the three sensors).

To investigate the level of uncertainty in the registration process, this procedure was executed on 20 different acquisitions with the plane rotated with different quantities and directions. The uncertainty was estimated by measuring the distance between the corners of the plane obtained from the 3 sensors after the registration process. There were 3 estimations of each corner of the plane, one for each sensor. The distance between these points, which referred to the same corner, was used to determine the uncertainty of the calibration procedure. This distance was estimated to be on average 16 mm, and the standard deviation 5.5 mm. These values are definitely below the range of displacements to be measured during the described wind tunnel test. At the same time, it should be noted that the measured quantity is the displacement of the structure, which does not depend directly on the registration procedure, since a large part of the structure displacement is not related to overlapping parts of the point clouds (Figure 5). A similar registration procedure is presented in [28], with an accuracy ranging a few centimeters.

A reconstruction of the tensile structure for both geometries can be seen in Figure 5. Only the points related to the object under inspection are visible since the background was removed through segmentation by considering the Euclidean distance of points (outliers’ removal [32]).

### 3.4. Tests Summary

To study the cover deformation due to wind exposure, the test was performed at different wind speeds and for different angles of incidence of the wind. The variation in the angle of incidence was obtained by rotating the structure that was linked to a rotating platform. A summary of the tests carried out is shown in Table 2. The wind velocities were related to the power percentage of the engines of the wind tunnel.

For each wind velocity and exposure angle, 30 depth frames for each sensor were acquired. Having set the sensor acquisition at 30 fps, the acquisition time was 1 s, for each wind velocity. Acquisitions were performed also for each angle with no wind.

### 3.5. Data Analysis Method

After the point cloud registration process, to obtain a measure of the displacement of the structure, it is necessary to find a relation between the point clouds acquired during the test and the ones before the tests, with no wind. 

**Figure 6:**
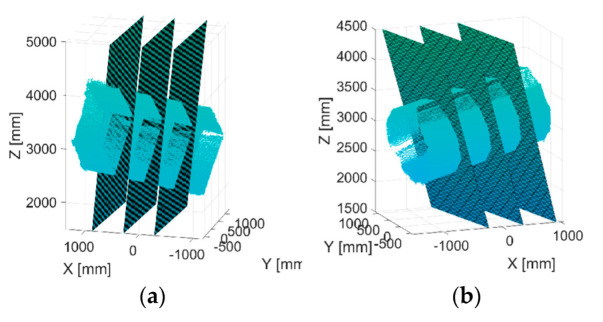
Planes to extract the sections of the structures of rectangular (**a**) and decagonal (**b**) structures.

**Figure 7:**
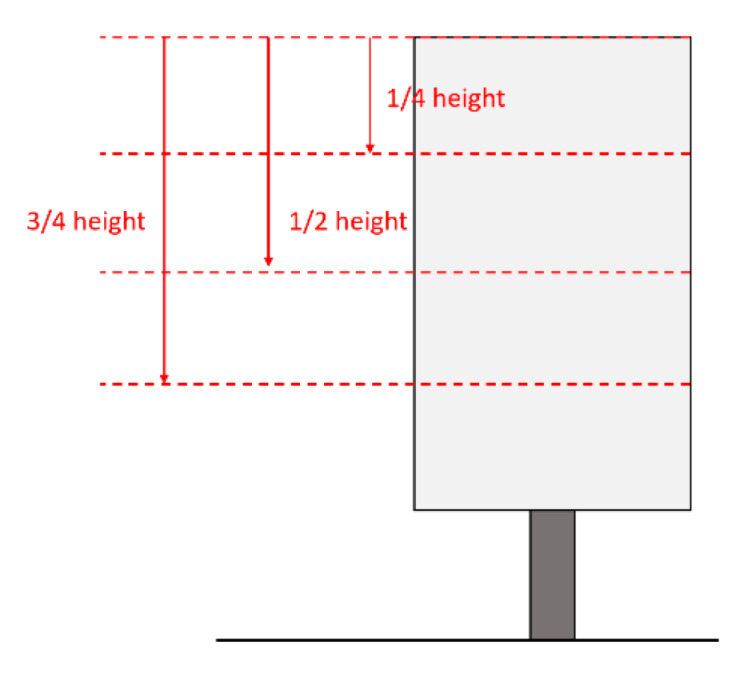
Schematic diagram showing the height of the sections.

To find this relation, 3 sections of the object were considered. These sections were defined by the planes shown in Figure 6. The points within 10 mm of distance from the sections were projected on the planes and a section of the object could be extracted. The position of each section compared to the height of the structure is shown in Figure 7. The planes of the sections were found by obtaining the sides of structure prisms and by computing the height from the basis of the structures on each side.

Point clouds of the 3 sensors after registration were defined with respect to the reference system of sensor 3. In particular, the edges of the squared and decagonal structures in the undeformed conditions were extracted from acquisitions of the structures not subjected to wind. After extracting the sections of the structures by projecting points within 10 mm distance from the sections, as described before, the sides of the structures for each section are given by fitting a straight line for each side. Using this procedure, the displacement can be computed as the point-line distance of the points of the deformed condition in relation to the straight lines of the undeformed condition, for each side.

**Figure 8:**
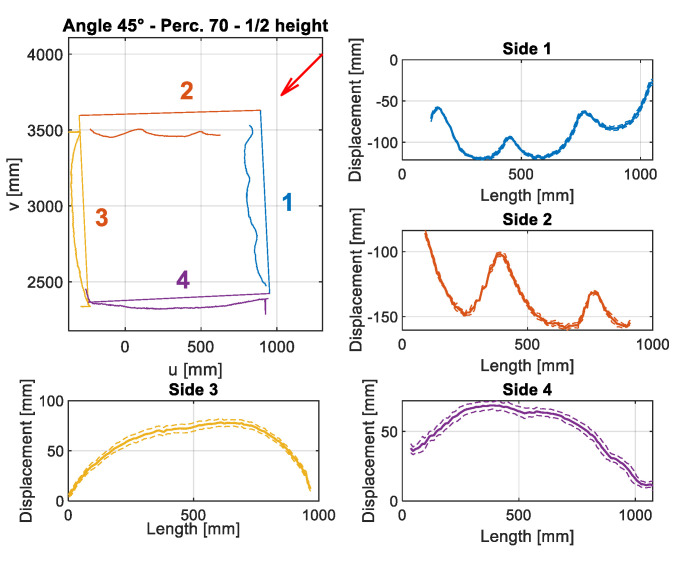
Example of the analysis performed for the squared structure of section at ½ of total height at wind speed of 158.5 km/h. The wind direction is indicated by the red arrow.

**Figure 9:**
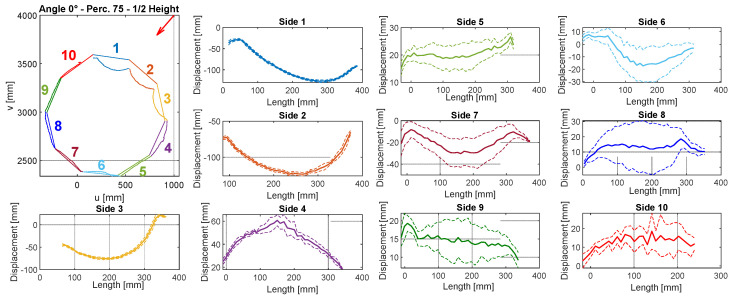
Example of the analysis performed for the decagonal structure of section at ½ of total height at wind speed of 176.5 km/h. The wind direction is indicated by the red arrow.

The final result of this procedure is the displacement calculated and displayed in graphs in Figure 8 and Figure 9 (similar graphs, but with different values, were calculated for each velocity and angle). The error source, which influences the displacement measures, is the uncertainty of the sensor, since the bias is deleted when the displacement is computed as the difference between deformed and undeformed object measurements. The second source of measurement error can be given by the point cloud registration to align the 3 sensors to the same reference system. The influence of the point cloud registration on the results has already been discussed in Section 3.3. Displacement is computed for each frame acquired and mean and standard deviation are extracted for each wind speed. For each side of the structure, the deformation trend is visible. In addition, the dashed line curves are defined, as the mean displacement ± standard deviation (Std. Dev.). Displacement is computed as the mean displacement for each millimeter along the length of the side. In order to avoid outliers, in particular at the corners of each side, in the case of a low number of points per millimeter along the length of the side, the average and standard deviation were not considered. This was done to reduce the noise related to error measurement and to discard unreliable data.

At the corners of the structure, the displacement can be subjected to mistakes due to possible errors of Kinect V2 sensors, described as mixed pixels errors, in [29]. On the other hand, the computed standard deviation is caused by both vibration and by sensor uncertainty. However, the sensor uncertainty can be quantified, since the sensor noise is given by the distance at which the object is framed. Since the object was placed between 2 and 3 m from the sensors, the uncertainty given by the sensors is expected to be about 2–3 mm, according to the sensors’ uncertainty [20]. Moreover, point cloud registration can have an impact, as described in Section 3.3. This level of uncertainty can be confirmed by lower standard deviation levels for the sides of the structures that are the most exposed to wind (sides 1-2 Figure 8 and sides 1-2-3 Figure 9). On these sides, the vibrations are almost negligible and the standard deviation is mainly caused by the sensor uncertainty. Moreover, it is difficult to split the points of the deformed structure section and relate them to the correct side of the structure, when they are close to corners. For these reasons, from displacement graphs, the corners of the structure are partially excluded. The 2D graph in Figure 8 that represents the squared structure refers to a single frame, while graphs are related to the entire acquisition for one single wind speed (30 frames). The 2D graph scales are related to the sensor 3 origin and they are the coordinates of the plane at ½ of the total height section. 

**Figure 10:**
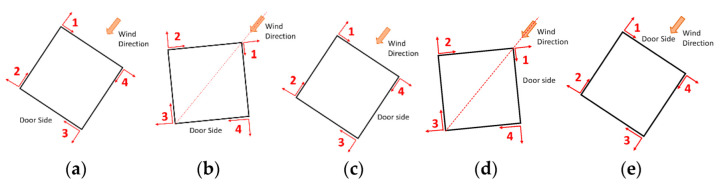
Reference systems for squared structure test for (**a**) 0°, (**b**) 45°, (**c**) 90°, (**d**) 135°, and (**e**) 180°.

**Figure 11:**
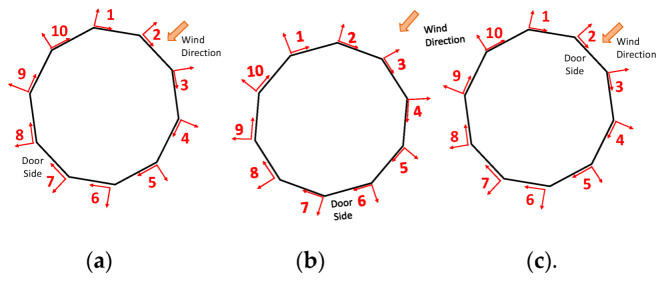
Reference systems for squared structure test for (**a**) 0°, (**b**) 45°, and (**c**) 180°.

The displacement of the graphs in Figure 8 and Figure 9 are referred to in the reference systems in Figure 10 and Figure 11. By convention, the reference exposure angle compared to the wind is defined as shown in Figure 10 and Figure 11. Positive displacement is always directed outwards and the reference systems are named in a clockwise direction. Reference systems are shown for each angle of the tests performed (Table 2).

By looking at the results in Figure 7 and Figure 8, it is possible to observe the symmetry between sides 1 and 2 in Figure 8, by noting the reference systems used to compute displacements for sides 1 and 2 in Figure 9. To observe this symmetry, one of the two graphs of the two sides should be flipped with respect to the center of the horizontal axis (length of the side). Regarding Figure 8, a symmetry can be observed between sides 1 and 3 with a reversed horizontal axis of side 1 or of side 3, since the reference systems are placed as in the scheme of Figure 10a.

To make a comparison between tests performed at different angles and wind speed, the average displacement of an entire side was computed.

## 4. Results

Using the procedure described before, the average displacement was calculated for each side, wind velocity, and structure section.

**Figure 12:**
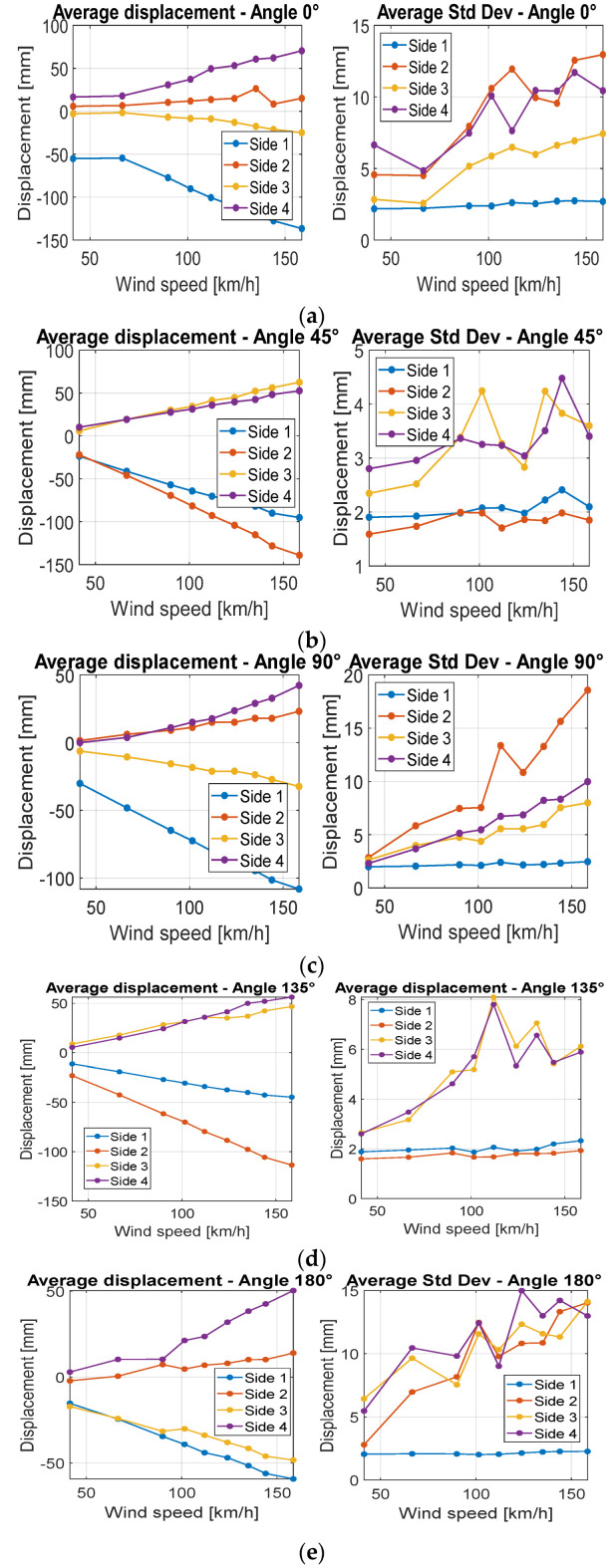
Average displacements and the standard deviation for each side of the squared base structure at 1/2 of the total height for different wind velocities for angles 0° (**a**), 45° (**b**), 90° (**c**), 135° (**d**), 180° (**e**).

**Figure 13:**
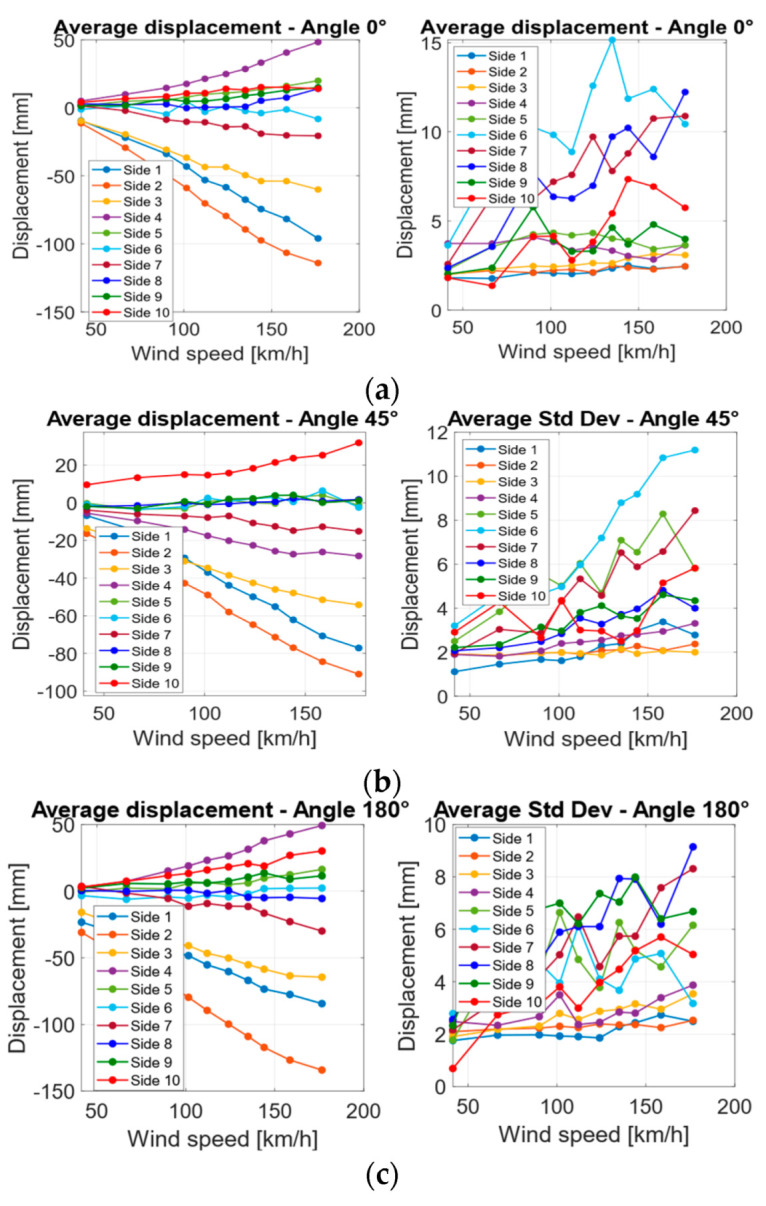
Average displacements and standard deviation for each side of the decagonal base structure at 1/2 of the total height for different wind velocities for angles 0° (**a**), 45° (**b**), 180° (**c**).

**Figure 14:**
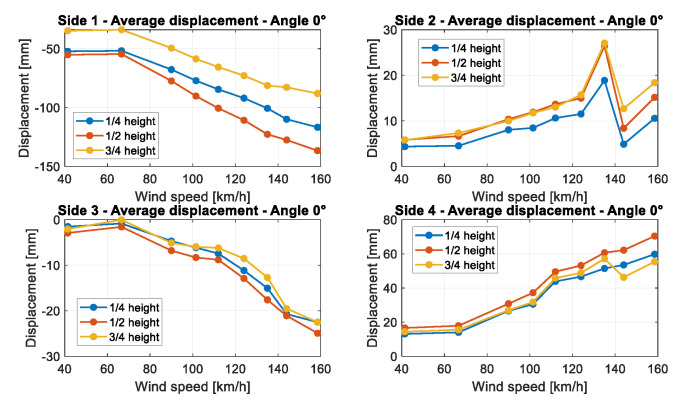
Comparison between average displacements at different heights of sections for the squared structure at 0° angle, for each side of the structure going from side 1 to 4.

In general, for both structures, tests evidenced the following results (Figure 12, Figure 13 and Figure 14):

1. The average displacement of the sides of the structure more exposed to wind changes approximately linearly compared to the increase in the wind velocity;
2. A higher average displacement and a larger vibration level were observed for the middle section with respect to the other sections, which are closer to the sides of the structure. This is reasonable since the central section is the farthest from steel borders that link the cover to the extremities of the object (Figure 6);
3. Greater displacements were measured at ¼ of the total length section with respect to ¾ of the total length section, since the ¼ section is more distant from the connection of the tensile structure to the ground (Figure 13);
4. The most exposed to wind sides are the ones that have the highest displacement and their adjacent sides are the ones subjected to the highest vibrations (standard deviation of displacement). This is likely because the wind first impacts against the sides more exposed and then it slides to the closest ones generating a separated unsteady flow, causing vibrations. This happens especially for the decagonal structure;
5. The displacements of the most exposed to the wind side and of the opposite side are directed inwards, suggesting an internal pressure lower than the external one, while the displacement of the other sides is usually directed outwards;
6. The most exposed to wind sides are the ones with the lowest vibration level;
7. Elastic belts connecting the two steel borders of the structure (Figure 1) can reduce significantly the average displacement when it is directed to the center of the structure, while they do not change the shape of the expected deformation when the displacement is outwards;
8. A higher displacement of the side most exposed to wind has a corresponding lower displacement of the opposite side.

The application of consumer 3D depth cameras, such as Kinect V2, is not a common practice for wind tunnel tests. However, the Kinect V2’s advantage is its low-cost, its wide FOV (suitable for large dimension objects 3D reconstruction), and its capability to acquire the point cloud at a frame rate of 30 fps or higher with a random error well below 10 mm [20] in the entire operative range. Thus, 3D reconstruction can be applied to measure the displacement of the entire structure, rather than perform measures of just a few points.

In this article, a method to measure the displacement of a structure in harsh environments, such as a wind tunnel, is presented. This method is based on the acquisition of three sensors’ point clouds. Since all the sensors remained in the same positions during the entire test, point cloud registration was performed only once before the test. The calculation of displacements was obtained by extracting three sections of the object. This procedure can be applied to the measurement of displacement for any structure, with a similar shape, for which a section can be extracted. 

One of the main advantages of this kind of measurement system is the application of low-cost and user-friendly sensors for 3D displacement measurements, in cases of a large deformation tensile structure. At the same time, this method does not require the installation of markers or speckles for optical measurements. Another advantage is the possibility to have the displacement of the entire structure under evaluation, given by the 3D shape measurements obtained for each point of the structure (with traditional measurement systems, it would be difficult to obtain such resolution in terms of displacement measures). On the other hand, a limitation of this procedure can be determined by the uncertainty of Kinect V2, which would be not suitable in cases of a low displacement of a structure. Indeed, these kinds of measurements require a sensor that has an uncertainty much lower than the expected deformation. The presence of edges makes it possible to locate the section to extract; in the case of irregular or very complicated geometry, the section to extract might be hard to detect.

The results allowed the behavior of the two structures starting from their deformed shape to be studied. Results at points 1 and 2 of Section 4 are expected and they allow the goodness of the measurement system to be checked.

It was possible to observe the shape of the structures for different speed velocities and angles. The average displacement and the standard deviation were computed since the results for these kinds of tests are related to a quantification of the displacement and 3D shape measurement of a structure when it is exposed to wind, in order to compare experimental results with numerical ones, for stationary wind conditions.

## 5. Conclusions

In this article, a measurement system involving the application of 3D cameras for a wind tunnel test is presented. The specific application of displacement measurement from a 3D shape is treated in this article and the analysis of acquired data allowed us to obtain the displacement of each side of the structures, for different sections. With reference to traditional measurement systems, this measurement system permits us to obtain the entire shape of the object being evaluated, and it is not measured only for specific points. As matter of fact, any section of the object could be extracted to determine the displacement. In comparison with other non-contact measurement systems, it does not require the application of markers on the object to measure. For these reasons, its application is suitable for the test described in this article. Other measurement systems, based on different 3D cameras, can be created with the same procedure of data analysis.

As a consequence, this measurement system can be seen as a complete system for measuring displacement, higher than the uncertainty of 3D sensors, and as a system for measuring the 3D geometry of structures in harsh environments. The frequency of vibration was not accurately measured in this experiment, since the purpose of the proposed technique was to measure the average displacement distribution of the 3D structure under test and even possible aliasing of the vibration would not affect the mean displacement. The measurement of the frequency of vibration can be part of future work, by acquiring sensors using an external trigger and by acquiring at an fps that is large enough to describe properly the vibrations of the structure (twice that of the vibration frequencies of the structure). Further improvements could follow these considerations involving the study of structural properties of structures with depth cameras, e.g., modal parameters or natural frequencies.

## Figures and Tables

**Table 1:** Kinect V2 characteristics [20].

Characteristic	Kinect V2
Working principle	Pulsed Time-of-Flight (ToF)
Depth Range	0.7–4.2 m
Max Depth Resolution	512 × 424
Max Color Resolution	1920 × 1080
Field Of View (FOV)	H: 70°, V: 60°
Max Acquisition Frequency	30 Hz
Latency time	20 ms
Software, SDK	Libfreenect2

**Table 2:** Tests summary.

Structure Type	Wind Velocities (km/h)	Wind Exposure Angles (°)
Squared base	41.4, 66.6, 90, 101.5, 112, 124, 135, 144, 158.5	0, 45, 90, 135, 180
Decagonal base	41.4, 66.6, 90, 101.5, 112, 124, 135, 144, 158.5, 176.5	0, 45, 180

## Data Availability

The data presented in this study are available upon request from the corresponding author.
